# From impossible to possible: the lessons from the control of recent COVID-19 outbreaks in China

**DOI:** 10.7150/ijbs.58906

**Published:** 2021-04-10

**Authors:** Lixin Tai, Kengieng Wong, Li Wang, Li-jun Di

**Affiliations:** 1 Cancer center, Faculty of health sciences, University of Macau; 2 Metabolomics core, Faculty of health sciences, University of Macau; 3 Institute of translational medicine, Faculty of health sciences, University of Macau

## Abstract

The COVID-19 pandemic has catastrophically impacted the world. Before the success in vaccination, this virus shows no sign of stop spreading. Nearly all the countries have implemented stringent approaches to slow down the transmission of the virus, but the virus still caused over 2 million deaths and the number is increasing. Therefore, preventing the virus spreading is still necessary to protect most people, especially the ones with pre-conditions. Mainland China has successfully eradicated the COVID-19 virus infection in Wuhan in 2020. After that, several small-scale outbreaks occurred in many cities in China, but none of these COVID-19 virus infections caused the widespread. In this review, we would like to give a detailed presentation of the approaches that were implemented by the China government to suppress the virus spreading by considering the unique characteristics of this virus and the paths of the virus transmission. Both the pros and cons of these strategies will also be analyzed. The experiences and lessons learned during the virus-fighting in China, expectedly, will be a useful source of reference for other regions in overcoming the threat caused by the COVID-19 virus.

## Introduction

COVID-19 has swept through the globe within a brief time and after one year; it is still continuously extending its territory. Millions of people are newly infected daily in the world. Several factors are important to contribute to the widespread of this virus. Fundamentally this virus is highly contagious. In some extreme cases, one patient could transmit this virus to over one hundred other people. To most of the victims, however, this virus only causes mild symptoms such as fever, cough, dizziness, etc. just like a regular cold without threatening life 1. So, most people simply serve as the virus carrier. The convenience offered by the modern transportation system, as well as the high amount of traveling population makes this virus spread all over the globe within a very short time 2. Recent studies, as well as the experiences from the small outbreaks in China where the initial large outbreak of COVID-19 in Wuhan has been eliminated, disclosed that this virus has unusual stability and survives a quite long time in the environment. When the north of the earth enters the winter season, the more and more live virus was found with the infectious ability on the surface of some objects including the already reported frozen food 3, 4. Therefore, many countries, while waiting for the vaccination of most of the people, still applied “social distance” and “isolation” strategies to slow down the virus spreading.

The pandemic caused by COVID-19 across the whole globe indicates that preventing the spread of this virus is nearly an impossible task. The reason is complicated because the virus could infect people in diverse ways. Also, the virus survives for a long time and there are too many non-symptomatic virus carriers too. Even whether wearing a mask will prohibit the dispersal of the virus is still an unsolved puzzle because the virus could be spread via a non-respiratory route. However, the outbreaks in China, including the Wuhan outbreak, have been contained and eradicated within few months. Now the Wuhan city is virus-free and the people regain the freedom to live a normal life, except wearing the mask as a precautious approach. Other cities such as Beijing and Shanghai have also experienced more than one wave of outbreaks of COVID-19 infection. Quickly, these infection events were under control and the infected individuals were quarantined on time. Frankly, the cost to suppress the spreading of the virus is not cheap. However, the benefits gained from the elimination of the virus are also valuable. Nevertheless, some valuable experiences were accumulated during the prevention of the virus spreading 5, 6. These experiences might apply to other less intensively influenced areas as well.

## Challenges for COVID-19 control

A common measurement for virus infection capacity is “R0”, or “R naught”, which shows how contagious a virus is by measuring the average number of people who contract the virus from one infected person. An R0 larger than 1 means the infection is exponentially expanding and restrictive measurements must be applied to contain the spreading of the virus. The estimated R0 for Covid-19 is diverse given by different studies with a range of 1.1 to 2 7. The R0 is also influenced by many factors such as the survival of the virus, the density of the population, how people responses to the virus, the hospitalization status, and the preventive strategies, etc. Ideally suppressing the R0 below a value of 1 is expected to decrease the number of cases after applying the approaches preventing the virus from spreading by the government 8. This explains why in many of the East-Asia regions such as Japan, South Korea, Hong Kong, etc., the number of new COVID-19 cases are relatively low.

### Viability of the COVID-19 Virus

The long-live ability of the COVID-19 virus has been recognized since the beginning of the pandemic. When the first outbreak of this virus in Wuhan in 2020, a study reported that the virus has unusual survival ability in the environment of the hospitals 9. Another study also examined the stability of the virus on different types of surfaces and found that the flat surface such as the auto parts, contains live virus after a few days 10. The stability of the SARS virus and COVID-19 in different conditions (aerosols, plastic, stainless steel, copper, and cardboard) using a Bayesian regression model has been compared and these two viruses showed similar half-lives in aerosols (1.1 to 1.2 hours) and on copper. Both of them show the most viability on the surface in stainless steel (5.6 hours) and plastic (6.8 hours), supporting that the transmission possibility through aerosol and fomite 11. A more striking finding by an Australian group is that the virus on the flat surface could survive for over 28 days at room temperature 12. Some scholars criticized that the experimental conditions applied by the Australian group are not like to be naturally occurred, in that the virus spread through the mucus or sweat which are hostile to the virus. But the detection of live virus from auto parts in several locations of China recently argues that the survival of the virus might be unexpectedly long. Also, both temperature and humidity have a considerable influence on the stability of the COVID-19 virus. A study performed 10 years ago by Lisa M. Casanova et al found that both low (20%) and high (80%) humidity protect the coronavirus much better than other conditions. The low temperature (4℃) could protect the coronavirus for over 28 days on the surface. Instead, the room temperature greatly reduces the survival of the virus 13. The recent outbreaks of COVID-19 at several locations in China were traced back to the frozen sea food or packed meat imported from other countries, which echos that low temperature and high humidiy protect the virus that contamiates the surface of the packages.

The initial outbreak of COVID-19 was in winter. People expected that when the summer comes, the pandemic might get less severe owing to that the virus might lose resistance to the high temperature, high humidity, and high UV, just like many infectious viruses such as SARS and influenza which are seasonal and become less transmissible as the climate warms. However, some studies showed that the transmission ability of COVID-19 was not sensitive to climate change. Data collected from 244 cities in China suggested that the basic R0 was not significantly associated with temperature changes and UV intensity 14, this has also been confirmed by the COVID-19 pandemics in tropical areas and countries, including Brazil, Ecuador. The same results were also reported by analyzing the data collected in New York, London, and Delhi according to a climate-dependent epidemic model 15. In short, unlike SARS, the warming of the climate will have mild or even no influence on the spread of the COVID-19 virus.

Disinfection of the environment and preventive hand sterilization was based on the fact that the virus can't survive the disinfectants or alcohol-based sanitizer. To test the effects of different disinfectants against the virus, the survival of the virus in different disinfectants such as bleach, hand sanitizer, ethanol, povidone-iodine, chlorophenol, chlorhexidine, benzalkonium chloride, etc. were analyzed. No infectious virus could be detected after 5 min treatment, except for hand sanitizers, which took a long time to kill the entire virus at room temperature. The team also found that the virus was very stable even at a variety of pH levels (pH3-10) and can still be detected in infectious concentrations for 60 minutes 16. This unique survival ability of the COVID-19 virus increases the difficulties to prevent its transmission. Also, this result reminded people that sanitizer might be not enough to get rid of the risk and washing hands frequently is necessary.

### The mechanism of COVID-19 invasion

The efficiency of human cell infection by the virus is critical for the spreading of the virus. ACE2 is a widely expressed protein distributing on the cell membrane and was identified as the major mediator of COVID-19 virus invasion. The mechanism of COVID-19 virus invasion of the human cell via binding to ACE2 has been studied in detail. After binding to ACE2, the spike (S) glycoprotein of the COVID-19 virus is cleaved into S1 and S2 subunit by Transmembrane protease serine 2 (TMPRSS2) and FURIN and then entry into the host cell. A single-cell mRNA sequencing from major adult organs revealed that ACE2 shows more expression in the kidneys and digestive system than in the lungs and trachea 17. In addition, hyperglycemia, a condition that occurred in the diabetes patient, increases the expression and the abnormal glycosylation of the ACE2 receptor, which may greatly increase the risk of COVID-19 infection 18. Diabetic patients are more susceptible to the COVID-19 infection and generally have a worse prognosis and higher mortality 19 20 21.

Compared with the SARS virus and influenza virus, COVID-19 is more contagious and infects more organs. Some other important receptors or co-receptors that bind to the S protein of COVID-19 in addition to ACE2 may exist. For example, a recent study found that Tyrosine-protein kinase receptor UFO (AXL) binds to the N-terminal domain of the S protein and co-localizes to the cell membrane. Knocking out AXL significantly reduces COVID-19 infection in lung cells. The expression level of AXL is closely correlated with S protein abundance in bronchoalveolar lavage fluid cells from COVID-19 patients 22. AXL is widely expressed in most of the organs in human and therefore might explain why the COVID-19 infection has a long-term negative effect on many parts of the body. Moreover, another potential COVID-19 receptor, neuropilin-1 (NRP1), has been revealed by two independent teams recently 23, 24. NRP1 expresses on the cell membrane and binds to a polybasic Arg-Arg-Ala-Arg carboxyl-terminal sequence on S1 subunit of S protein. Blocking NRP1 with small-molecule inhibitor or monoclonal antibodies efficiently decreases the infection ability of the COVID-19 virus too. The existence of these non-ACE2 mediators of COVID-19 invasion of human cells increases the difficulties in preventing the virus infection, vaccine design, and the treatment of patients.

### Non-symptomatic carrier

The abundance of the non-symptomatic carrier of the COVID-19 virus causes more trouble to prevent the spreading of the virus. Asymptomatic infection refers to those who have no relevant clinical manifestations, such as fever, cough, sore throat, and other self-perceived or clinically recognizable symptoms and signs, but the respiratory tract and other samples tested positive for the virus. As early as February 2020, half of the people infected with the COVID-19 virus on the Diamond Princess cruise ship in Japan were found to be asymptomatic 25. In another example, an immunologically compromised woman was found to be asymptomatic for over 100 days and the shed viruses were potentially infectious for at least 70 days 26. A high proportion of asymptomatic infection cases have been found in many regions including mainland of China 27. Roughly half of the virus-infected people show no or very mild symptoms and can be classified as asymptomatic cases. Asymptomatic infections have been found in people covering multiple age groups, even children 28. Although asymptomatic patients have no symptoms or have mild symptoms, the amount of virus released by these people is equivalent to that of patients with symptoms 29. Also, the asymptomatic patients are lacking self-perceived clinical symptoms and go through a long incubation period with the ability to infect. Only through the rigorous virus test, these virus carriers will be found and quarantined as did in China. These potentially risky people were quarantined in the so-called “Fang Cang” hospitals. This strategy has become the most important approach to interrupt the virus transmission during the fighting against the COVID-19 virus in Wuhan, China.

## Transmission of COVID-19

The recognition of the multiple ways for the COVID-19 transmission was based on the experiences accumulated over a long time of battle against this virus. The strategies to trace the virus transmission and to interrupt the virus spreading are also based on the detailed understanding of the virus transmission mechanisms.

### Droplets transmission

Similar to other coronaviruses such as SARS-CoV and MERS-CoV, the COVID-19 viral particles mainly infect the human respiratory tract 30. Virus carrying droplets or aerosols are considered the primary infectious agents produced by patients through coughing, sneezing, talking, and breathing 31, 32. The contagious droplet, of which the diameter over 5μm, is large enough to directly fall into the air and reach the mucosa, nasal or oral cavity of the others if the social distance is less than one meter 33, 34. The smaller infectious droplets or aerosol ≤5μm in diameter can disseminate into the atmosphere for longer and travel further (more than one meter apart) from the origin to the susceptible people, which is defined as airborne transmission 35. Shreds of evidence showed that the COVID-19 virus RNA lingered in the air for up to 16 hours 36 and the virus remained viable in the air for 3 days using laboratory aerosolization method according to another report 37. Some clinical studies also reported that the COVID-19 virus dispersed and remained viable in the air sample of enclosed hospital wards 38, 39. Besides, a huge number of cases reported that indoor crowded and inadequately ventilated settings such as choir practice 40, 41, densely populated sports facilities 42, nursing home 43 also caused COVID-19 infection clusters. Therefore, preventing human gathering and contact is the most efficient way to slow down the spreading of the virus.

### Fomite transmission

Fomite transmission can occur by touching the COVID-19 virus-contaminated objects unintentionally and therefore is believed to be another potential route to spread the virus. Clinical reports discovered that environmental samples of COVID-19 wards were identified with viral RNA 44, 45, and a laboratory experiment showed that the virus can maintain certain viability on object surfaces up to 48hr 37. Several small-scale outbreaks occurred recently in China showed that the COVID-19 virus can spread through the objects. For example, the Xinfadi, Beijing outbreak was started by the staff, which had no contact history of the COVID-19 virus, working with frozen seafood. The only possibility for the infection is through the frozen seafood, which was tested to be virus-positive 3, 4. The strain of the virus is a European variant, suggesting the re-emergent of the virus might be due to the imported goods 4. In the following months, several cases related to direct or indirect contact with contaminated frozen food, food packaging materials, or cold-chain storage were reported 46, 47. Also, several people responsible to deliver the food to the COVID-19 patients under quarantine were found to be infected without direct contact with the patients. The mediator of the infection could be through the food packages. Although The World Health Organization and the Centers For Disease Control and Prevention of the United States claimed that the risk of being transmitted COVID-19 via handling or processing contaminated frozen food is not high 48, 49, preventive measures should be enforced for the cold-food chain workers.

### Fecal-oral route

To increase the accuracy in deciding if a COVID-19 patient is no longer infectious, and to find the virus carrier in the close contacts of the confirmed COVID-19 cases, the anal swab was also implemented in addition to the oropharyngeal swab in controlling the recent outbreaks in China. The reason is that the COVID-19 could infect the gastrointestinal (GI) epithelial cells. The virus RNA was detected in patients' stool 50-52 and urine 53. The ACE2 receptor has wide expression in the small intestine, kidneys, lung, colon, liver, etc 54. Importantly, environmental samples such as sinks, toilet bowls 44, and drainage system 55 have been detected with virus RNA, which suggests viral shedding in feces can be a probable route of transmission. It also draws public attention to fecal aerosol vertical transmission via dried-up U-traps in high-risk apartments 55. Also, sewage overflow and sewage-contaminated aerosols could cause virus spreading in the urban region 56. To prevent the virus spreading through the fecal-oral route, the personal hygiene and detoxication of the environment have to be implemented carefully.

### Ocular route of transmission

Since the SARS outbreak in 2003, it has been believed that coronavirus can transmit via direct or indirect contact with the mucosa of the eyes 57. With short physical distance, virus-contaminated aerosol or droplets can easily land on the conjunctival epithelium and spread to the upper respiratory tract since the two parts are connected by the nasolacrimal duct 58. In the early outbreak of COVID-19 in Wuhan of China, a physician got infected in the hospital and he was wearing an N95 mask and other personal protective equipment but no goggles to protect his eyes 59. A few COVID-19 patients developed ocular manifestations such as conjunctivitis and conjunctival hyperemia and were confirmed virus RNA positive in ocular samples 59-61. Laboratory research demonstrated that inoculation of the virus in ocular conjunctiva can cause mild COVID-19 symptoms in rhesus macaques 62. Hence, COVID-19 transmission through eyes must not be neglected and eye protection is necessary, especially for health-care workers 63, 64. Also, cleaning the hand by washing or sanitizing are efficient ways to avoid the virus infection via ocular route.

### Maternal-to-fetal transmission

The risk of a pregnant woman getting COVID-19 is not higher than others but it draws public attention to COVID-19 virus transmission from infected mothers to the fetus. A few clinical cases reported that vertical transmission is rare (2 of 31 COVID-19-positive pregnant women) but possible 65. ACE2 is highly expressed in specific cell types of the maternal-fetal interface 66 and neonatal congenital infection of COVID-19 could occur by virus from maternal blood going through from placenta to cord blood, which solely belonged to fetus. Congenital COVID-19 phenotypes including immune cell infiltration in the placenta 67 and neurological manifestations in the neonate 68 have been reported. Of note, COVID-19 specific IgM was found in neonates born to a COVID-19 mother 69, 70. Unlike IgG, IgM cannot transfer across the placenta due to its large molecular structure, which indicates the infants were infected in utero and transplacental transmission is contingent to happen. However, the maternal-to-fetal transmission generally causes no harm to the public.

### Zoonotic transmission

The reservoir hosts of COVID-19 are generally considered as bats since sequence analysis showed it shared high nucleotide homology with SARS-like coronavirus isolated from Chinese horseshoe bats 71-74. How the virus transmitted from bats to humans and its intermediate hosts is still unknown but one study identified COVID-19-related coronavirus in Malayan pangolins caught in anti-smuggling operation in China 75. Also, COVID-19 had been identified in domestic pets from households with COVID-19 patients 76, 77, the tiger in the zoo 78, and farmed minks in the Netherlands and Denmark 79-81. Lassaunière R., et al. found that the new spike mutation showed up in Danish farmed minks and transmitted to humans 82, ending up all minks were culled across the country 81. Therefore, the possibility of the virus transmission through the animals cannot be excluded. Precautions should be paid to avoid the animals, especially the pets, to carry the virus and transmitted it back to humans.

## Approaches to prevent the virus from spreading in China

While the spreading of COVID-19 virus shows no sign of slow down before the widely vaccination globally, the Mainland China has achieved zero local new case for nearly a half year after the Wuhan outbreak. Although the new cases still continuously appear in several different places later, China has developed the strategies to eradicate these infections locally. The experiences accumulated during these fights against COVID-19 are valuable but may not be applicable to other places owing to complicated reasons.

### Zero infection policy

With the largest population in the world and with the first and a large-scale outbreak of the COVID-19 pandemic in Wuhan, China quickly suppressed the spreading of the virus and achieved the zero-local new case on March 19 of 2020. Afterward, some small-scale outbreaks appeared in several cities especially the ones close to the border to other countries. Nearly all these outbreaks were under control and achieved “zero” new case finally. The benefit of the “zero” policy is that once there is any new case reported, the path of the virus transmission can be determined through the epidemiological investigation. In addition, when there is no new case, the community becomes safe and the normal economic activities can be resumed.

However, such a goal can only be achieved when there is a border to control the flow of people. China has enforced border control when the pandemic becoming severe across the globe 83. After the local infections were eradicated, China implanted even more stringent border control because several small-scale breaks of virus infection were caused by the cross-border travelers. Within China, although completely cut off the domestic travel is impossible, some very stringent polices were implemented to prevent the local travelers. Each local government set up border check at the highway entrance, the airport, and the train stations to identify any suspected virus carriers by registering the travelers with their health declaration, body temperature and virus test result. For example, the Zhuhai city has implemented these measurements at the beginning of the Wuhan pandemic and identified 76 COVID-19 patients without causing any community spreading 84, 85. Macau, a neighbor international city of Zhuhai with a half-million population, has also enforced its border control since the COVID-19 pandemic started 86. Most of the cases associated with Macau were travelers who have been under quarantine without bringing risk to the local community. The very few local cases were found at the beginning of the pandemic and all were cured in the hospital. Thus far, Macau has maintained the “zero new case” for nearly a year. Not only in mainland China but New Zealand also achieved “zero” new infection starting from May 2020 87. However, such a record was broken by international travelers later. Since this island country continues to apply approaches such as rigorous virus testing, prolonged quarantine, efficient contact tracing, etc., the appearance of new cases is kept at an exceptionally low level without the risk of a large-scale community outbreak. However, low levels of local spreading of the virus still generate negative impact to the economic activities.

**Figure 1:**
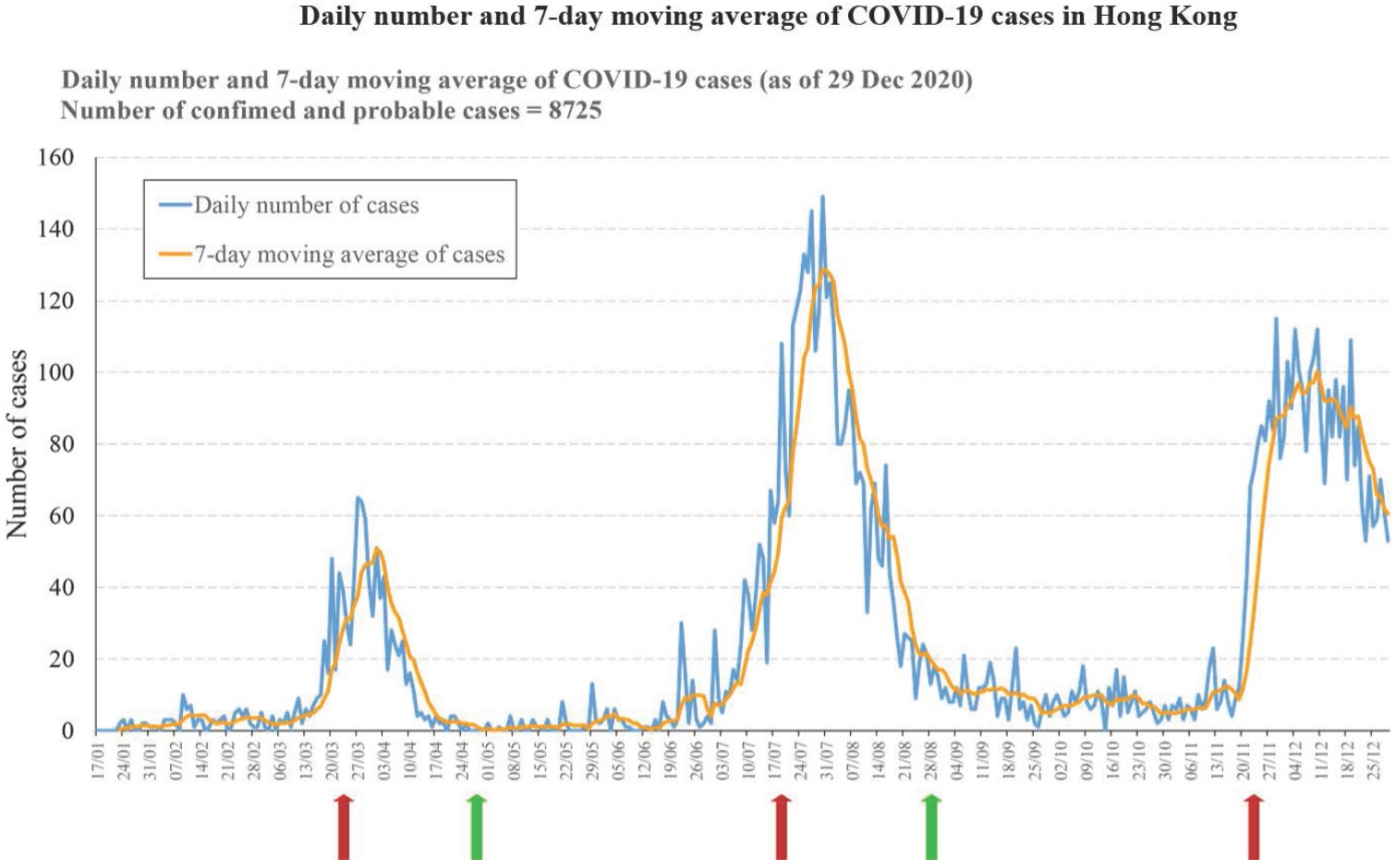
Daily change of COVID-19 virus positive cases in Hong Kong. The diagram shows the daily number of COVID-19 cases in Hong Kong since Jan 17, 2020. Three virus infection peaks were observed within 2020. The arrows indicate the implementation of regulations on social distancing by Hong Kong Government. Red arrows indicate the implementation time point of the stringent regulation and green arrows indicate the releasing time point of the regulation. The implementation of the stringent control of social distancing is followed by a peak of infections. The releasing of the social distancing control at the end of each infection peak is followed by a long period of low new cases. Of noting is that the third wave of infection contains two peaks without change of the social distancing restriction policy.

In comparison, the pandemic of COVID-19 may persist for a much longer time in places without enforcing the “zero” policy. For instance, Hong Kong has achieved low infections from April to June 88. However, with the release of the restriction on social activities, the number of infections started to increase in July. When the restrictions are applied again, the cases start to drop again. From September to November is the second flat period with a small number of new cases daily. However, the average case number over this period is significantly higher than the average case number over the period after the first peak, suggesting the virus is spreading at an accelerated rate. In December, another even bigger outbreak started, and another round of restriction has to be implemented. However, the 3^rd^ wave of virus spreading sustained much longer and become more devastating. Immediately after the Chinese New Year, the Hong Kong government issued the restriction releasing policy again in anticipating that the forthcoming vaccination may reduce the risk of community transmission of the virus. After all, the stringent restriction on social distancing in Hong Kong lasted for around 2 months in total and results in over 190 deaths during the COVID-19 pandemic. If the policy was more stringent and follows the rule of 14 days no new case before releasing the restriction, Hong Kong may have the chance to eradicate this virus at the first hand (Figure 1).

### Rigorous virus checking for high-risk population

Rigorous virus test in the population with high-risk of contracting COVID-19 virus is enforced in mainland China. For example, all the people who work in high-risk fields such as the public transportation department, the hospitals, the farmer's market, and the airports, etc. must perform the routine virus test. The bus drivers and taxi drivers, who have a higher chance to be exposed to the virus carriers, were required to perform the virus test on a routine basis. All the teachers also need to do the virus test before the school reopens in early 2020. The rigorous virus test ensures the early detection of virus carrier and safeguards the suppression of the further virus spreading. The most recent example is the identification of the new cases in Shanghai. Three non-symptomatic cases were identified in a round of routine virus tests for the contractors of one of the major hospitals 89. Through the epidemiological investigation, more cases were identified by tracing down the close contacts. Similarly, one new case was identified in Pudong airport from the workers in the luggage department 90. Then more close contacts were traced down and quarantined. Successfully, the Pudong outbreak was eradicated eventually.

A major way of infection, according to the recent small-scale outbreaks, was caused by the imported goods or international travelers 91. Thus, the people who participate in the transportation, testing, and distribution of the imported goods, or the quarantine and transportation of the international travelers, have to do the virus test weekly while they are also required to wear the personal protection equipment (PPE). With this policy, several small-scale infections by either the imported goods or the international travelers were spotted early which greatly reduced the chance of expanded infections.

### Early identification of virus carrier

In the mainland of China, a frequent practice in the hospitals is that the patients with fever, if seeks for clinic diagnosis, must be screened for COVID-19 test first. Even in the emergency department of all the hospitals, if the visitor has a temperature above 37.3, the patient is asked to do the COVID-19 test before any immediate treatment or seeing any doctor 92. Since most of the cities follow this guideline strictly, the outbreaks in several cities were under control because the early cases, when having symptoms, will seek a clinical diagnosis in hospitals where they are identified as the COVID-19 patient. Tracing of “close contacts” of the confirmed cases will automatically be started. This alerting system has been critical for limiting the virus spreading and eventually leads to the eradicating of the virus infections. For example, the first case from the Xinfadi market outbreak was identified when the patient visit hospital for treatment of cold-like symptoms in Beijing 93. The most recent outbreak in Shijiazhuang, Hebei, however, showed how much damage the virus could cause if the identification of the early cases were failed 94. When the first non-symptomatic patient was confirmed to be COVID-19 positive in this city, the virus has already been spreading in the rural area of Shijiazhuang for several weeks. The reason is that the people living in a rural area lost the vigilance to the virus. Many people chose to ignore the mild symptoms caused by COVID-19 because the flu is also popular in winter in north of China. So, these COVID-19 positive patients were treated by the local clinics in their neighborhood without quarantine or virus test. In fact, the clinics in rural region became a center for virus transmission among patients because many people share the same room for regular treatments. Comparing to other small to middle-scale outbreaks in Dalian, Shanghai, Beijing, etc., the failure to identify the earlier victims leads to the exponential expansion of the virus infection in Shijiazhuang.

### Social distance

One meter of social distance has become a “golden standard” to prevent virus transmission in places where there is a gathering of people 64. The effectiveness of this measurement is also based on the precondition that everybody wears the mask. Some places require more than one meter to further reduce the risk of virus transmission. These guidelines are critical to prevent the wide spreading of the virus among people, but still not enough. To achieve the “zero” transmission, mainland China has implemented much more stringent control over the social distance. For example, to successfully control the Wuhan pandemic, most citizens were asked to stay home. In China, people live in the apartment complex which is equipped with its own entrance control. The people who do not live in the apartment complex, are also organized to form subdistricts separated by streets. These apartment complexes or subdistricts were required to set up entrance control. So, the in-and-out of the residents was stringently prohibited. The government has organized the delivery team to send the essential living materials such as the grocery and medicine etc. directly to each household. When the situation is getting better, each family was allowed to have a limited number of passes for each day to fulfill their living needs. In the rural region, people are under strict control from leaving or entering the villages too. In recent days, the Gaocheng district of Shijiazhuang is marked as a “high risk” area. So the whole district has to be locked down. In fact, the subway in the whole Shijiazhuang city was also stopped to prevent the travel of people across the city, suggesting enforcing the social distance and prohibiting the flow of people is an effective way to prevent the spreading of the highly contagious virus.

Even during the Chinese New Year (CNY), when the pandemic is still at its peak in 2020, most people chose to give up visiting each other. In fact, the government extended the CNY holidays to encourage people to stay home. In addition, all the public areas were closed including the restaurant, theaters, parks, etc. Even the public transportation was canceled for a brief period in cities heavily hit by COVID-19. Since the virus takes less than 2 weeks to cause symptoms in most people, the sharp and stringent lockdown breaks the transmission of the virus and gives people a time window to identify and quarantine the infected ones. In comparison, most other countries rely on law and self-obedience to keep the social distance. For example, the UK policy allows people to leave their homes with a necessary excuse 95. Such flexibility allows people to live an easy life during the pandemic. But the risk for virus spreading is also increased owing to the inevitable people gathering.

### Big Data supported Health declaration

Tracing the travel route of individuals is illegal in most countries including China. However, when the vaccine is not available and the COVID-19 virus is highly contagious and sometimes lethal, it is necessary to prevent the potential risk as early as possible. For example, when no new COVID-19 case was reported starting from March 19, 2020, after the Wuhan outbreak, many areas in China have started to resume the normal. To reduce the risk, both the central and the local governments implemented the health declaration QR code to identify the individuals with potential risk. The QR code is based on the recording of the travel route of the cell phones carried by nearly everybody 96. For example, the QR code has three assorted colors with green as no risk, yellow as low risk, and red as high risk. Most of the places including the parks, libraries, theaters, buses, and restaurants, etc. require the QR code before entering. All the cell phones, if in use and connected to the service network, will have their time-dependent location information recorded. This information is uploaded to the central server in real-time which is the basis to decide the color of the QR code. If by any chance a COVID-19 positive patient is confirmed, the travel route of this patient can be outlined based on the record. Then all the cell phones that have shared the same cell tower at the same time will be classified and the risk level will be raised. For example, the cell phone might start to show a yellow QR code. In addition, the confirmed patient must provide the details of his travel route which helps to further identify the potential contacts. The people who had a chance to get too close to the confirmed case, shared the same doorknob, entered the same address at the same time, took the same public travel facilities, etc., will be found and their health declaration code will be in red. This big data-based strategy ensures that the virus can be traced down and the transmission route can be outlined and stopped. In fact, this strategy worked so well that many people were quarantined and tested for the positive virus infection even before they show any symptoms or realize that they had been exposed to the viruses.

### Extended quarantine of high-risk travelers and the follow-up checking after releasing

China has applied restrictions to cross-border activities since the COVID-19 pandemic outbreak. Initially, all the international flights were canceled. When the domestic pandemic was under control, some international flights were resumed. However, all the routine flights facing a potential risk of one-week suspension if there are COVID-19 positive passengers on that flight 97. More than that, the passenger has to present the negative report for both COVID-19 and the anti-COVID-19 antibody. After the flight landed, all the passengers will be transported to the appointed hotel for quarantine for 14 days 83. Shortly the 14-day quarantine policy was extended to 21-day owing to the recent observation of the “re-positive” cases who have been released after the 14-day quarantine. The same policy applies to all the international passengers including the Hong Kong travelers to mainland. In fact, owing to the continuous extending of local infections in Hong Kong, the number of people crossing the Hong Kong/Shen Zhen Custom has been significantly reduced.

### Mobile cabin hospital

When the infection accelerates, more victims are found. However, many of these infected individuals show no symptoms, or the symptoms are relatively unharmful. These people are advised to stay home for self-quarantine in many countries. This has resulted in infections among family members. Inevitably, these infections also leak to the community later. Starting from the Wuhan outbreak, all the infected individuals, no matter with or without symptoms, will be quarantined in the facilities provided by the government in China 98. Even in the hospital, the patients with positive COVID-19 have to be separated from the non-infected area. When the infection is in a smaller scale, the patients are accepted into the hospitals with quarantine facilities. If the patient number exceeds the capacity of hospitals, the government will construct mobile cabin hospitals to accept the patients with severe symptoms. At the same time, the large public facilities such as the sports complex, hotels, meeting halls, etc. will be transformed to separate units, known as “Fang Cang hospital”, and to host the patients with minor symptoms. During the recent outbreak in the rural area of Shijiazhuang, the people from 3 heavily infected villages were transported to other quarantine facilities. A 14-day quarantine of these people ensures that the virus transmission can be interrupted. The villages will be disinfected after all the people are moved out. Since the outbreak occurred in winter, this strategy solved the problem of potential contamination by the long-lived virus in the environment too.

### Precisive epidemiological investigation (PEI)

**Figure 2:**
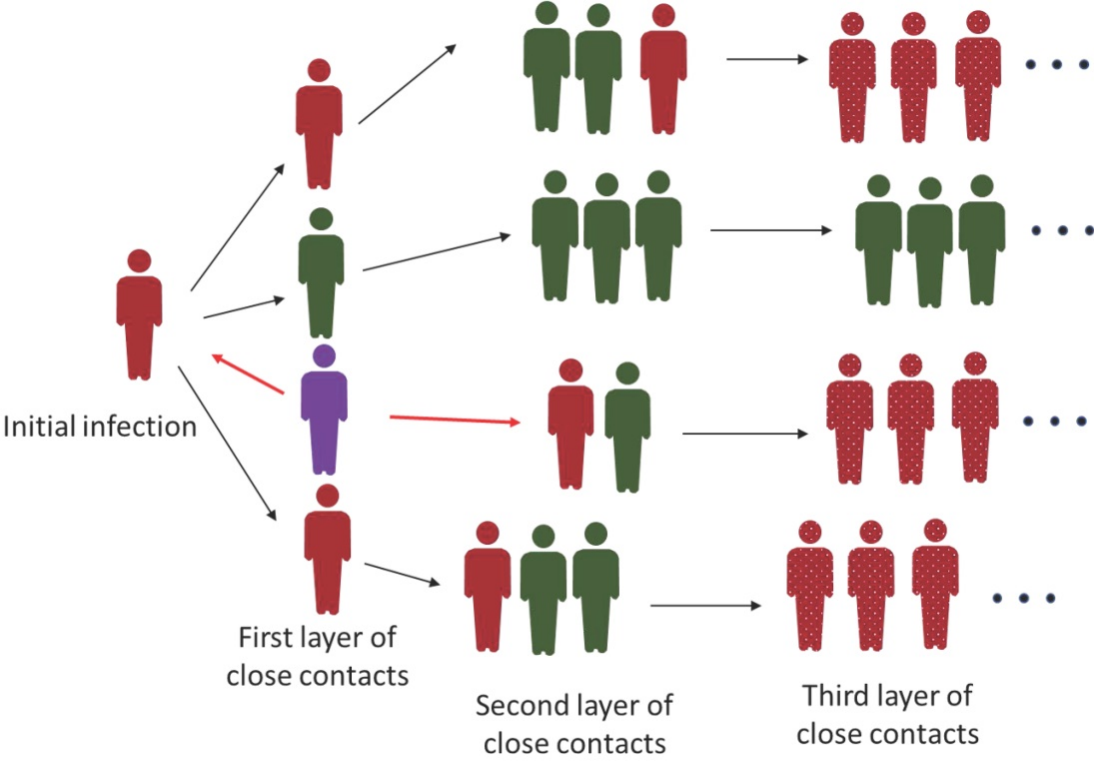
Interruption of the transmission of COVID-19 virus by PEI. The diagram describes the way of the COVID-19 virus transmission and how the PEI is applied to interrupt the virus spreading. Generally, the awareness of the first positive case as indicated as purple individual here, is through routine virus test. Of noting is that this first case may not be the initial case. From the purple case, both the upstream and downstream close contacts will be found and quarantined through PEI (red arrow). PEI will be further applied to identify the second and sometimes even third layer of close contacts as indicated as black arrows. Green cases represent the close contacts with negative virus test and red cases represent the positive contacts. The green cases will also be quarantined unless the upstream close contact is confirmed to be virus negative. The dotted red cases indicate the undetermined number of infected patients after the third layer of close contacts which could be a large number.

To deal with the recent outbreaks in several cities of China, the cities chose to lock down the whole city to screen for the positive virus carrier or COVID-19 patients. The lockdown often associates with the “freezing” of all the people movement. This strategy has been efficient to make the Wuhan virus outbreak under control because there was a large number of infections and active transmission of the virus. However, when only a limited number of cases were identified in a city, implementation of the lockdown causes inconvenience to all the residents if the government can't support the least needs of the people efficiently. Also, the tedious virus test for all the people is not economical. Therefore, if a city has equipped with a good epidemiological investigation team, implementation of PEI to find the people potentially infected by the virus is a cost-effective way. For example, the small-scale outbreaks in Shanghai, a city with a nearly 20 million population, were managed via PEI 99. By investigating the travel route, determined based on the big-data analysis and personal interview, the close contacts of the confirmed cases will be found and quarantined. The second or even third layer of close contacts will also be found, and these people will be restricted and isolated until the upper level of close contacts is confirmed to be negative for virus infection (Figure 2). The PEI strategy has the risk of virus leaking owing to the missing of key information. So, the condition to apply this strategy is that there is only a limited number of confirmed cases and all the related people can be cooperative to supply the correct information. Once the outbreak starts to expand exponentially and extensively, the PEI will be inefficient to catch up with the spreading of the virus. That's why there is no PEI implemented in countries with heavy COVID-19 infections.

## Perspective

COVID-19 is so far the most devastating infectious disease on a scale similar to the Spanish flu in human history. However, the challenge brought by this disease is even worse because of the modern interconnected economic activities globally. Thus, the destruction caused by this virus is also severe in human history. In addition to the over 2 million deaths caused by this virus, the global economy was impaired fundamentally. Most of the countries have their economies shrink except the mainland China with a slowdown of economic growth. The reason is simply that China earned more than half a year of virus-free time which allows the restart of the economic activities. In addition, the rest of the world has been deeply involved in the wrestling with the virus and has increased demand for the products from China, even more than the amount they used to have because many countries lose the normal ability to be as productive as before under the virus defending circumstances. Since China has been one of the major manufacturers for the global market before the “COVID-19” crisis, the successful elimination of the virus in early 2020 gave China a significant opportunity to further increase its productivity and expand its share of the international market.

The COVID-19 firstly broke out in Wuhan China. Afterward, the virus quickly swept most of the other countries. Owing to insufficient knowledge about this virus, most of the countries did not take immediate action to prevent the virus invasion. China has learned from the Wuhan disaster quickly and applied strict isolation and quarantine approaches to contain the virus from spreading. However, the people who live in those cities that were locked down experienced some challenging difficulties. In few cases, people suffered the sacrifice of life that is not directly caused by the virus but by the lockdown. But the payback is cutting the pain of suffering to be short. With the price of fewer than 100,000 cases of infection and 5000 deaths, China has eradicated the virus within 2 months. Importantly, several smaller-scale outbreaks were also successfully eliminated without causing any deaths.

The total cost for the COVID-19 pandemic was 25 billion USD in China till 31th May 2020 100. However, considering China has gained an increase in the economy growth by defeating the COVID-19 pandemic within a brief time, the benefit from economy growth should have defrayed the cost of defeating COVID-19 pandemic. Among the 25 billion USD spending, only 0.2 billion US dollars were spent to treat the COVID-19 cases till the middle of 2020 in China. For the in-patient treatment, each case costs over 6000 USD according to a study 101. All the cost was mainly afforded by the government. A large portion of the money was spent to contain the virus, to do the virus test, to establish quarantine of the infected patients, to compensate the people who lose income caused by the lockdown of the cities, to support the least needs of the people who are required to stay home, to support the people who are in-duty or simply volunteered to fight the virus, etc. However, the allocation of the resources including the financial support by the central government may not be applicable by other countries owing to the different legislation. Also, the total cost incurred in other countries by the virus is also hard to be estimated because the cost could be divided by different parties that are involved, including the commercial insurance, health care providers, the government and each individual.

Conclusively, mainland China has become a unique example of fighting the COVID-19 virus. Some of the strategies are applicable to other countries too. For example, the rigorous virus test is an cost-effective and easy-way to identify the risky non-symptomatic virus carriers. The establishment of the “Fang Cang hospital” is also an applicable approach to prevent the community spreading of virus. However, this approach may not be easily acceptable because some people may consider the hospitalization is not deserved with minor symptoms. Some of the strategies implemented by China may not be suitable for other countries because of the different infrastructure of the government, clinical organizations, and culture, etc. For example, the tracing of the cell phone is forbidden by many countries because it may violate the human rights. The implementation of such an approach has to be protected by the central government as China is able to do. Similarly, the PEI approach may violate the personal rights as well. In fact, several people infected by COVID-19 have become victims owing to the leaking of personal information during PEI, although such information leaking is strictly prohibited by law.

After all, it is difficult to tell whether the approaches implemented by China are better than the other approaches implemented by other countries. But if simply analyze the impact on the economy, the change of the life of the people and the deaths caused by COVID-19, China has won more and lose less.
